# Supplementary material for: A Critical View of the Application of the APEX Software (Aqueous Photochemistry of Environmentally-Occurring Xenobiotics) to Predict Photoreaction Kinetics in Surface Freshwaters
Source: Molecules. 2019 Dec 18;25(1):9. doi: 10.3390/molecules25010009 (PMC7017383; doi:10.3390/molecules25010009)
Supplement: Supplementary file 1 [file molecules-25-00009-s001.zip › 3.2.4_gcc-4.4.0.zip!/3.2.4_gcc-4.4.0/3.2.4_gcc-4.4.0/doc/octave/html/interpreter/Evaluation.html]

Evaluation - Untitled


Next: Statements,
Previous: Expressions,
Up: Top


---

## 9 Evaluation

Normally, you evaluate expressions simply by typing them at the Octave
prompt, or by asking Octave to interpret commands that you have saved in
a file.

Sometimes, you may find it necessary to evaluate an expression that has
been computed and stored in a string, which is exactly what the
`eval` function lets you do.

— Built-in Function: **eval** (try, catch)  
> Parse the string try and evaluate it as if it were an Octave
> program. If that fails, evaluate the optional string catch.
> The string try is evaluated in the current context,
> so any results remain available after `eval` returns.
>
> The following example makes the variable a with the approximate
> value 3.1416 available.
>
> ```
>           eval("a = acos(-1);");
> ```
>
> If an error occurs during the evaluation of try the catch
> string is evaluated, as the following example shows:
>
> ```
>           eval ('error ("This is a bad example");',
>                 'printf ("This error occurred:\n%s\n", lasterr ());');
>                -| This error occurred:
>                   This is a bad example
> ```

- Calling a Function by its Name- Evaluation in a Different Context
